# Characterisation of immunoparesis in newly diagnosed myeloma and its impact on progression-free and overall survival in both old and recent myeloma trials

**DOI:** 10.1038/s41375-018-0163-4

**Published:** 2018-06-20

**Authors:** Jennifer L. J. Heaney, John P. Campbell, Gulnaz Iqbal, David Cairns, Alex Richter, J. Anthony Child, Walter Gregory, Graham Jackson, Martin Kaiser, Roger Owen, Faith Davies, Gareth Morgan, Janet Dunn, Mark T. Drayson

**Affiliations:** 1 Institute of Immunology and Immunotherapy, University of Birmingham, Birmingham, UK; 2 University of Bath, Bath, UK; 3 University of Warwick, Warwick, UK; 4 University of Leeds, Leeds, UK; 5 University of Newcastle, Newcastle, UK; 6 Institute of Cancer Research, London, UK; 7 St James’s University Hospital, Leeds, UK; 8 University of Arkansas for Medical Sciences, Little Rock, AR USA

## Abstract

We measured immunosuppression at myeloma diagnosis and assessed the impact on survival in 5826 UK myeloma trial patients. Polyclonal immunoglobulin levels were below normal in 85% of patients and above normal in only 0.4% of cases for IgA, 0.2% for IgM and no cases for IgG. Immunoparesis had a greater impact in recent trials: median overall survival (OS) was up to 3 years longer for patients without immunoparesis compared to the old trials, less than 1 year longer. Median progression-free survival (PFS) was 39%, 36% and 57% longer for patients with normal IgG, IgA and IgM levels, respectively. The depth of IgM suppression, but not the depth of IgG or IgA suppression, was prognostic for survival: the most severely suppressed IgM tertile of patients OS was 0.9 years shorter than those in the top tertile, and 2.6 years shorter than OS of those with normal IgM levels (*p* = .007). The degree of suppression of polyclonal IgM levels below normal was associated with worse PFS (*p* = .0002). Infection does not appear to be the main mechanism through which immunoparesis affects survival. We hypothesise that IgM immunoparesis impacts through a combination of being associated with more aggressive disease and reduced immune surveillance against relapse.

## Introduction

Multiple myeloma (MM) is an incurable neoplastic disorder that arises from the proliferation of a single clone of plasma cells in the bone marrow and accounts for over 10% of haematological malignancies [1, 2]. An established feature of MM is suppression of the adaptive immune system and resultant low levels of polyclonal antibodies (immunoparesis) [3–8]. This T and B lymphocyte immunosuppression increases the susceptibility to both bacterial and viral infections, and the antibody levels have been shown to be significantly lower in myeloma patients who experienced serious infections [9–11]. Infection is a major cause of morbidity and mortality in myeloma patients, particularly in the first 3 months from diagnosis [10, 12, 13], with immunoparesis, a contributory factor to this that we are currently investigating in a UK trial of antibiotic prophylaxis (TEAMM). Less attention has been given to longer-term outcomes and the hypotheses, that increasing depth of immunosuppression is a result of more aggressive MM (thus a marker of poor prognosis) and separately that immunosuppression renders MM more likely to progress because of the reduced immune surveillance.

Immunoparesis and an associated increased risk of progression to active MM occurs in 20% of MGUS patients and 70% of smouldering MM [14 –17]. In active MM prevalence, low antibodies increased from 63% in Durie-Salmon stage I to 90% in stage III [4]. This was also found in a recent registry-based study in 1755 consecutive myeloma patients over 22 years to 2012, available through the Greek Myeloma Study Group [18]. At least one isotype was suppressed in 77% versus 88% versus 94% of patients with International Staging System-1, -2 and -3 disease, respectively. Preservation of the uninvolved immunoglobulins was found in 13% of patients, which was associated with significantly longer overall survival. This study did not include clinical trial patients, but in a subset of 500 patients, there was sufficient follow-up data to assess progression-free survival, which was longer for patients with normal polyclonal antibody levels. This group found immunoparesis commoner in patients with IgA MM, while a previous study found immunoparesis commoner in IgG MM [5]. These studies did not compare the depths of immunoparesis for different antibody isotypes, which may be important, given the different anatomical sites of normal plasma cells secreting polyclonal IgM (lymph nodes and spleen) versus IgG and IgA (bone marrow). In addition, previous studies have not investigated the significance of suppression of the alternate polyclonal free light chain. Thus, there remains a need to investigate the significance of the depth and character of immunoparesis in the context of modern anti-myeloma therapy with detailed long-term survival outcomes to explore the potential consequences of increased risk of infection, reduced tumour surveillance and association with poor prognosis of MM.

To further characterise the prevalence and severity of immunoparesis at diagnosis and the prognostic significance for overall survival (OS) and progression-free survival (PFS), we have retrospectively analysed data from a large cohort of 3247 newly diagnosed MM patients enroled in the Myeloma IX and Myeloma XI clinical trials. To compare the impact of immunoparesis on survival outcomes between modern anti-myeloma therapy and pre-biological treatment regimes, we also assessed the relationship between immunoparesis and survival in 2807 patients from the MRC myeloma trials from 1980 to 1997, prior to the establishment of novel agents and the benefit of intensive therapy.

## Methods

### Patients and clinical trials

Patients were enroled in either the MRC Myeloma IX (MIX) trial (ISRCTN68454111) or the Cancer Research UK Myeloma XI (MXI) trial (ISRCTN49407852), and henceforth will be referred to as the ‘recent myeloma trials’. In these multicentre, phase III trials, newly diagnosed patients were divided between an intensive and a non-intensive pathway based on their eligibility for the autologous stem cell transplantation and assessed in relation to progression-free and overall survival (PFS and OS). MIX randomised to receive thalidomide versus non-thalidomide-containing therapy, thalidomide could be given both as an induction and/or as a maintenance regimen [19–22]. MXI compares lenalidomide with thalidomide induction therapies and assesses the value of bortezomib in poor responders. In remission, patients are randomised to no maintenance or to receive lenalidomide, or lenalidomide–vorinostat maintenance therapy. Data were available for 3247 patients from MIX and MXI (up to a randomisation date of 1 June 2013 to allow for sufficient patient follow-up). However, only the data on 3218 out of the 3247 patients, where polyclonal IgM was recorded as part of the central laboratory immunochemistry analysis, were included in the patient characteristics table and multivariate analysis. To compare these trials using current anti-myeloma therapy to trials pre-dating novel biological agents, data were included from 2807 patients who enroled in MRC myeloma trials (MIV, MV, MVI and MVIII) from 1980 to 1997, henceforth referred to as the ‘old’ myeloma trials. Treatment allocation within these trials has been described previously [23]. The patient characteristics table and multivariate analysis were performed on 2608 of 2807 patients, where IgM levels were recorded. Multicentre research ethics committees and local ethics committees approved all the trials, and all patients gave written informed consent.

### Measures and patient classification

Serum from diagnosis was analysed centrally by protein electrophoresis, densitometry and immunofixation for M-protein quantification and characterisation. Serum IgG, IgA and IgM, and kappa and lambda free light chains (sFLC) levels were quantified by turbidimetry. Patients were characterised as having one of the following multiple myelomas: IgG, IgA, IgM, IgD, light chain only (LCO), non-secretory (NS) or oligosecretory; with either kappa or lambda monoclonal light chain. Patients were classified as being below, within or above normal range (NR) for polyclonal immunoglobulins based upon 5th–95th centile ranges of adults aged over 45 years in the UK reported by PRU (Protein Reference Units): IgG 6–16 g/L; IgA 0.8–4 g/L; and IgM 0.5–2 g/L. For sFLC levels, patients were classified as being above, within or below NR (3.3–19.4 mg/L for lambda and 5.7–26.3 mg/L for kappa) [24].

Patients were classified into two groups according to the presence or absence of adverse cytogenetic abnormalities: standard risk (no adverse cytogenetic abnormalities) and high risk (one or more adverse cytogenetic abnormalities). Adverse cytogenetic abnormalities were defined as gain (1q), t(4;14), t(14;16), t(14;20) or del(17p) [22, 25].

### Statistical analyses

Immunoparesis at diagnosis was assessed for patients in MIX and MXI trials. Mann–Whitney *U*-tests and Kruskal–Wallis tests were used for comparisons between M-protein groups and kappa and lambda myelomas, as appropriate. Spearman’s rank correlations were performed to evaluate the relationships between M-protein and polyclonal immunoglobulins, and sFLC. Data were analysed using IBM SPSS statistics version 21.

Patient characteristics are presented for old and recent myeloma trials by polyclonal IgM (within NR, below NR). Differences in patient characteristics were investigated using the Pearson’s *χ*^2^-test with continuity adjustment used wherever appropriate. Survival curves were constructed using the method of Kaplan and Meier, and the log-rank test was used to assess the differences between the groups, in the old myeloma trials and recent myeloma trials [26, 27]. OS was defined as ‘date of entry to the trial’ to ‘date of death’ or censored at ‘last alive date’. PFS was defined as time from date of entry to progression or death, or censored at date last known to be alive and progression free. Progression was defined as relapse from complete response, if one was achieved or documented progressive disease [28]. Survival outcomes were analysed between patients who were below or within the NR for polyclonal immunoglobulins and FLCs, and assessed based on the degree of immunoparesis. Degree of immunoparesis was obtained by splitting the patients who were below the NR into three groups for IgG, IgA and IgM individually. Cox regression was performed to determine whether polyclonal IgM was an independent predictor of OS and PFS after adjusting for Sβ2M, BMPCs and ISS in the old trials, with the addition of LDH and genetic risk in the recent trials [29]. Survival analysis and Cox regression were performed using SAS statistical software (SAS Institute, SAS Circle, Cary, NC, USA).

## Results

### Patient characteristics

The study included 2608 patients older MRC trials (21% with polyclonal IgM within NR and 79% with polyclonal IgM below NR) all receiving conventional therapy as described previously, Table 1 [23]; and 3218 patients from recent trials (11% with polyclonal IgM within NR and 89% with polyclonal IgM below NR). The proportion of patients aged ≥65 years increased from 50% in the old trials to 56% in recent trials, reflecting an increase in patients ≥65 years receiving intensive therapy. Twenty-four per cent of patients were ISS stage I in recent trials compared to 9% in older trials reflecting improvements in diagnosis.

**Table 1:** Characterisation of patients included in the present analyses by immunoparesis of polyclonal IgM (within normal range versus below normal range) from those enroled in MRC myeloma trials from 1980 to 1997 (old trials) compared to the MRC Myeloma IX trial (2003–2008) and the ongoing NCRI Myeloma XI trial (2010–2016)

		Old trials (MIV, MV, MVI and MVIII)				Recent trials (MIX and MXI)			
Factor	Grouping	IgM within NR *N* = 535	IgM below NR *N* = 2073	Total *N* = 2608	*p*	IgM within NR *N* = 348	IgM below NR *N* = 2870	Total *N* = 3218	*p*
Gender	Male	298 (56%)	1196 (58%)	1494 (57%)	.45	198 (58%)	1645 (59%)	1843 (59%)	.77
	Female	236 (44%)	876 (42%)	1112 (43%)		144 (42%)	1148 (41%)	1292 (41%)	
Age group	<65 years	287 (54%)	1018 (49%)	1305 (50%)	.07	169 (49%)	1226 (43%)	1395 (44%)	.05
	≥65 years	248 (46%)	1051 (51%)	1299 (50%)		179 (51%)	1632 (57%)	1811 (56%)	
Pathway	Intensive	–	–	–	–	210 (60%)	1627 (57%)	1837 (57%)	.21
	Non-intensive	–	–	–	–	138 (40%)	1243 (43%)	1381 (43%)	
M-protein type	IgG	343 (64%)	1131 (55%)	1474 (56%)	.001	231 (66%)	1700 (59%)	1931 (60%)	.002
	IgA	123 (23%)	580 (28%)	703 (27%)		60 (17%)	708 (25%)	768 (24%)	
	IgD	4 (1%)	36 (2%)	40 (2%)		4 (1%)	51 (2%)	55 (2%)	
	IgE	1 (<1)	1 (<1)	2 (<1%)		–	–	–	
	Light chain only	55 (10%)	296 (14%)	351 (13%)		43 (12%)	370 (13%)	413 (13%)	
	Non-secretory	9 (2%)	29 (1%)	38 (1%)		7 (2%)	17 (1%)	24 (1%)	
	Oligosecretory	–	–	–		3 (1%)	24 (1%)	27 (1%)	
% BMPCs	<20	152 (40%)	331 (21%)	483 (24%)	<.0001	189 (61%)	774 (29%)	963 (32%)	<.0001
	20–50	156 (41%)	724 (45%)	880 (44%)		99 (32%)	1182 (45%)	1281 (43%)	
	>50	76 (20%)	559 (35%)	635 (32%)		23 (7%)	699 (26%)	722 (24%)	
Sb2m (mg/l)	≤4	208 (40%)	460 (23%)	668 (26%)	<.0001	235 (68%)	1317 (46%)	1552 (48%)	<.0001
	4–8	195 (37%)	807 (40%)	1002 (39%)		89 (26%)	1069 (37%)	1158 (36%)	
	>8	117 (23%)	765 (38%)	822 (35%)		23 (6%)	480 (17%)	503 (16%)	
Albumin (g/l)	<30	79 (22%)	458 (29%)	537 (27%)	.001	55 (17%)	559 (20%)	614 (20%)	.008
	30–35	106 (30%)	535 (33%)	641 (33%)		81 (24%)	846 (30%)	927 (30%)	
	>35	174 (48%)	612 (38%)	786 (40%)		195 (59%)	1397 (50%)	1592 (51%)	
eGFR ml/min	<30	89 (17%)	453 (22%)	542 (21%)	.0003	19 (6%)	287 (10%)	306 (10%)	.008
	30–60	230 (43%)	961 (46%)	1191 (46%)		116 (34%)	995 (36%)	1111 (35%)	
	>60	215 (40%)	654 (32%)	869 (33%)		207 (60%)	1502 (54%)	1709 (55%)	
ISS	I	81 (17%)	146 (7%)	227 (9%)	<.0001	134 (41%)	608 (22%)	742 (24%)	<.0001
	II	169 (36%)	617 (32%)	786 (33%)		128 (39%)	1086 (40%)	1214 (40%)	
	III	214 (46%)	1189 (61%)	1403 (58%)		67 (20%)	1024 (38%)	1091 (36%)	
LDH IU/L	<273	–	–	–		92 (36%)	892 (42%)	984 (41%)	.07
	≥273	–	–	–		163 (64%)	1224 (58%)	1387 (59%)	
Genetic risk	Standard risk	–	–	–		115 (33%)	798 (28%)	913 (28%)	<.0001^a^
	High risk	–	–	–		43 (12%)	694 (24%)	737 (23%)	
	Unknown					190 (55%)	1378 (48%)	1568 (49%)	

^a^Test is based on those with a result only

Comparison of factors between groups with IgM within the NR and IgM below the NR in the older trials and recent trials demonstrated an increase in BMPCs and Sβ2M in IgM below NR groups compared to the within NR group, *p* < .0001, Table 1. Albumin was higher, where IgM was within the NR in both old and recent trials (*p* = .001 and *p* = .008, respectively). LDH recorded in the recent trials did not significantly differ between groups (*p* = .07). Patients with lower IgM were more likely to have adverse cytogenetic abnormalities present compared to those with IgM within NR (24% versus 12%, *p* = < .0001).

### Characterisation of immunoparesis

**Fig. 1:**
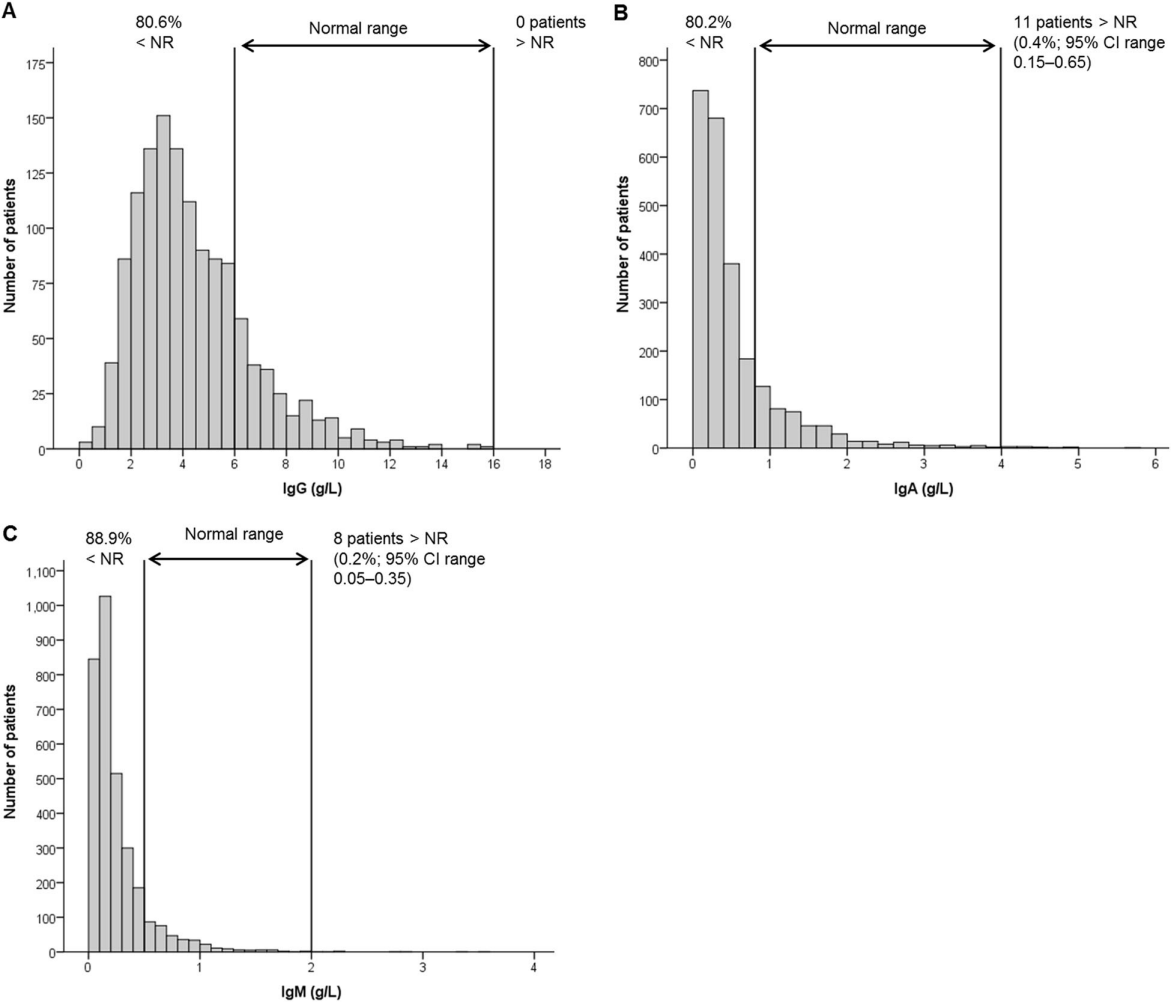
Levels of polyclonal immunoglobulin in MIX and MXI clinical trials. Data are presented for levels of polyclonal IgG (**a**, *n* = 1302), IgA (**b**, *n* = 2469) and IgM (**c**, *n* = 3226) at disease presentation in all myeloma patients without an IgG, IgA or IgM M-protein, respectively. For patients above normal ranges (NR), number of patients, percentage of patients and 95% confidence interval (CI) ranges for the percentage of patients are shown

Figure 1 illustrates the distribution of polyclonal IgG, IgA and IgM levels in patients without an IgG, IgA or IgM M-protein, respectively. IgM levels were below the NR in 89% of patients compared to 80% of patients for IgG and IgA levels. Median polyclonal levels (IQR) for IgG, IgA and IgM were 3.9 (2.7–5.6), 0.3 (0.2–0.6) and 0.2 (0.1–0.3) g/L, respectively. These values represent a reduction below the median value of the NR of 65% for IgG, 87% for IgA and 84% for IgM. No patient without an IgG M-protein had an IgG level above the NR of 16 g/L. A small proportion of patients presented with IgA (0.4%) and IgM levels (0.2%) above the NR; median (IQR) concentrations in these patients were 4.4 g/L (4.1–4.8 g/L) and 2.48 g/L (2.1–3.2 g/L), respectively. Individuals who were above the NR for IgA included five IgG patients, five LCO patients and one IgM patient. Patients who were above the NR for IgM comprised six IgA and two IgG patients.

### Immunoparesis by myeloma M-protein type

**Fig. 2:**
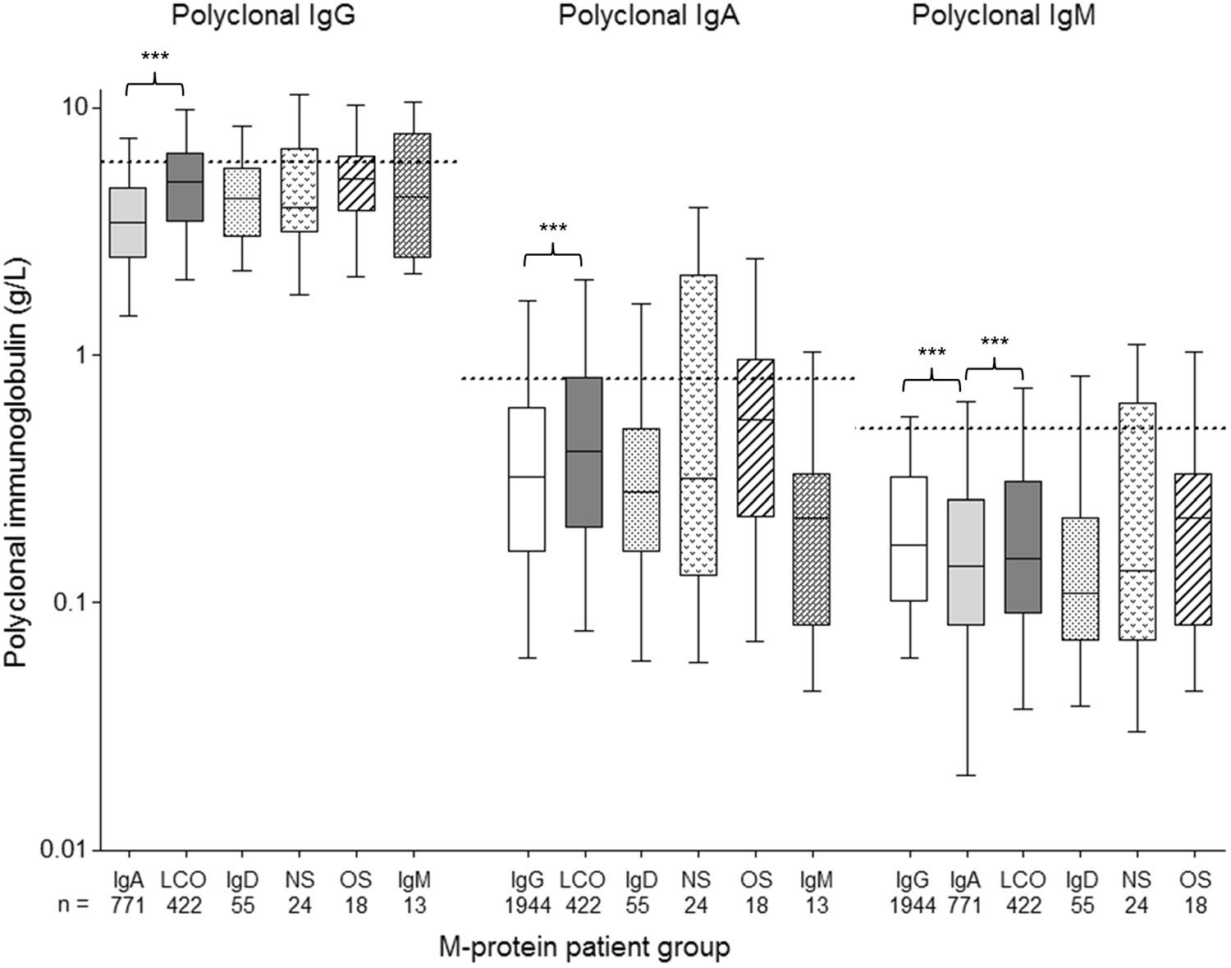
Levels of polyclonal immunoglobulins by the three main patient M-protein subgroups in MIX and MXI. Statistics were performed for the three main patient groups with sufficient sample size (IgG, IgA and LCO). Significant differences between groups are indicated, ***p < 0.001. Dotted lines indicate the lower limit of the normal range of serum polyclonal immunoglobulin concentration: 6 g/L IgG, 0.8 g/L IgA and 0.5 g/L IgM

Polyclonal immunoglobulin levels based on myeloma M-protein type are presented in Fig. 2. Group differences in immunoparesis were evaluated between the three main patient M-protein groups (IgG, IgA and LCO) who together made up 95% of all patients. IgA patients had the most profound immunoparesis, followed by IgG and then LCO patients, but differences between the M-protein types, although statistically significant, were not substantial (Fig. 2). Similarly, lambda patients demonstrated significantly but not substantially, lower polyclonal immunoglobulins across IgG, IgA and LCO patient groups (Supplementary Figure S1). Statistics were not performed for the remaining patient groups (IgD, IgM, NS and oligosecretory myeloma) due to small sample sizes; however, IgD patients had the lowest median polyclonal levels of all M-protein groups. This may be due to the high prevalence of lambda light chain type in IgD myeloma.

### Inverse relationship between M-protein level and polyclonal immunoglobulin levels

Higher M-protein levels were associated with significantly lower levels of polyclonal immunoglobulins. The strongest negative correlation was seen for IgA M-protein and polyclonal IgG (*r*_s_ = –.414). The amount of IgA M-protein also negatively correlated with the level of polyclonal IgM, *r*_s_ = –.253. In patients with IgG myeloma, negative relationships were also found between M-protein level and polyclonal IgA (*r*_s_ = –.271) and IgM levels (*r*_s_ = –.254). These relationships are illustrated in Supplementary Figure S2 (*p* < 0.001 for all correlations).

### Inverse relationship between M-protein level and polyclonal free light chain levels

The level of polyclonal sFLC (uninvolved immunoglobulin) was inversely correlated with whole IgG or IgA M-protein level (Supplementary Figure S3, *p* < 0.05 for all correlations). These plots of polyclonal sFLCs versus M-protein illustrate that Freelite did not identify polyclonal kappa sFLC levels between 2.5 and 5.5 mg/L and polyclonal lambda patients between 1.5 and 3.5 mg/L. To overcome this problem, we analysed kappa and lambda sFLC levels in a sub-cohort of IgG and IgA patients using monoclonal anti-free light chain reagents on a Luminex platform [30], which enabled a more sensitive assessment of the distribution of polyclonal sFLC levels without gaps in measurement (Supplementary Figure S4). These produced correlations of the same direction and strength compared to Freelite, with the exception of IgA lambda patients, where a stronger relationship was observed using Luminex data (*r*_s_ = –.406, *p* = 0.0001) compared to Freelite (*r*_s_ = –.126, *p* = 0.03). As the assessment of polyclonal sFLC was more precise with the Luminex assay for measurement below the NR, we used the platform to later assess the relationship between sFLC immunoparesis and survival outcomes.

### Survival outcomes by M-protein type

Survival data were available for 2587 patients from the old trials and 3109 from recent trials. Patients with IgG myeloma survived the longest in the old trials (median 2.52 years), followed by IgA myelomas (median 2.33 years) with LC-only myeloma patients doing worst (median 1.94 years, *p* < .0001, Supplementary Table 1). IgG patients continue to survive longest in recent trials and median OS has increased from 2.52 years in the old trials to 4.75 years. Light chain-only myeloma patients also benefited from increased OS (median 4.32 years in recent trials compared to 1.94 years in the old trials) and PFS (median 1.92 years in recent trials compared to 1.46 years in the older trials).

### Immunoparesis is associated with poorer survival outcomes

Patients from the recent trials survived longer than patients from the old clinical trials, median OS 4.53 years (95% CI = 4.32–4.84) and 2.33 years (95% CI = 2.23–2.46), respectively, despite a greater proportion of patients being older in the more recent trials. In both old and recent myeloma clinical trials, patients with immunoparesis at diagnosis had significantly poorer PFS and OS compared with patients for whom polyclonal immunoglobulin levels were within the NR (*p* < 0.01 for all comparisons, Supplementary Table 2). The association of immunoparesis with poor survival was stronger in recent than old trials. In the old trials, individuals with normal levels of polyclonal IgG had a median OS that was 19% longer than the OS of individuals with low IgG; in the recent trials, this OS benefit for individuals with normal levels of polyclonal IgG at diagnosis was 80% longer than in those with immunoparesis. For polyclonal IgA levels, OS was 30% and 39% longer and for IgM 29% and 50% longer in old and recent trials, respectively.

In the recent trials, median OS (95% CI), in years, for normal (19% of patients) versus low (81% of patients) polyclonal IgG was 6.93 (CI 4.89–7.33) and 3.84 (3.44–4.37), respectively; for normal (19% of patients) versus low (81% of patients) polyclonal IgA levels was 6.15 (5.21–7.33) and 4.42 (4.15–4.82), respectively; for normal (11% of patients) versus low (89% of patients) polyclonal IgM levels was 6.59 (5.29–8.08) and 4.37 (4.14–4.64), respectively (all *p* < 0.0003; Supplementary Table 2). In the recent trials, median PFS, in years (95% CI), for normal versus low polyclonal IgG levels was 2.41 (1.96–3.12) and 1.73 (1.62–1.86), respectively; for normal versus low polyclonal IgA levels was 2.49 (2.03–2.79) and 1.83 (1.73–1.93), respectively; for normal versus low polyclonal IgM levels was 2.83 (2.28–3.17) and 1.80 (1.72–1.89), respectively (all *p* < 0.0001; Supplementary Table 2).

Immunoparesis of the uninvolved sFLC (either kappa or lambda) did not impact upon OS or PFS; notably over two-thirds of patients had polyclonal sFLC levels in the NR, which in part reflects the high prevalence of reduced glomerular filtration in myeloma, itself an adverse factor for survival (Supplementary Table 2).

### The depth of IgM suppression, but not the depth of IgG or IgA suppression, is a prognostic factor for survival outcomes

**Fig. 3:**
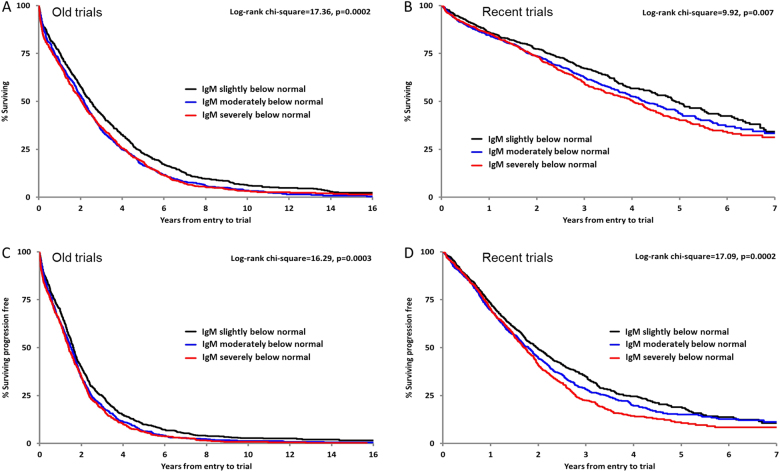
Survival outcomes in relation to degree of IgM immunoparesis. Overall survival by degree of immunoparesis of polyclonal IgM levels for old trials (**a**) and recent MIX and MXI trials (**b**). Progression-free survival by degree of immunoparesis of polyclonal IgM levels presented for old trials (**c**) and recent MIX and MXI trials (**d**). Patients below the normal range for polyclonal IgM (<0.5 g/L) were divided into three tertiles based upon degree of suppression: slightly below normal >0.2 to <0.5 g/L; moderately below normal >0.1 to ≤0.2 g/L; severely below normal <0.1 g/L

In the recent MIX and MXI trials, median OS was 6.59 years in the 342 patients with normal polyclonal IgM levels at diagnosis and 4.37 years in the 2855 patients with IgM levels below the NR at diagnosis. Dividing these 2855 patients into tertiles, median OS in the lowest IgM tertile group was 4.02 years compared to 4.26 years in the middle group and 4.92 years in the tertile with the least severe immunoparesis (*p* = 0.007; Table 2 and Fig. 3). Examining the depth of immunoparesis by tertiles for polyclonal IgG and IgA levels at diagnosis showed no significant difference at *p* = 0.98 and 0.65, respectively. The same was found in the old trials, where the greater the depth of IgM immunoparesis, the shorter the median OS, whereas the depth of IgG or IgA immunoparesis was not associated with altered OS (Table 2).

**Table 2:** Overall survival (OS) and progression-free survival (PFS) for patients by the degree of immunoparesis

		Old trials (MIV, MV, MVI and MVIII)				Recent Trials (MIX and MXI)			
	Grouping (g/L)	*N*	Median (95% CI)	Censored	Log-rank x^**2**^, ***p***	*N*	Median (95% CI)	Censored	Log-rank x^**2**^, ***p***
OS									
Polyclonal IgG levels (for non-IgG myeloma)	>4 to <6	288	2.22 (1.85–2.57)	5%	0.55, .76	367	3.84 (3.25–4.90)	53%	0.05, .98
	≥3 to ≤4	243	2.02 (1.65–2.31)	7%		287	4.02 (3.00–4.53)	54%	
	<3	249	1.98 (1.62–2.39)	4%		389	3.69 (3.30–4.50)	55%	
Polyclonal IgA levels (for non-IgA myeloma)	>0.3 to <0.8	509	2.42 (2.17–2.68)	5%	3.69, .16	832	4.30 (3.86–4.96)	57%	0.86, .65
	≥0.2 to ≤0.3	627	2.05 (1.86–2.17)	5%		402	4.90 (4.02–5.44)	59%	
	<0.2	322	2.29 (2.01–2.63)	5%		734	4.29 (3.94–4.96)	58%	
Polyclonal IgM levels (for non-IgM myeloma)	>0.2 to <0.5	689	2.48 (2.21–2.72)	7%	17.36, .0002	918	4.92 (4.49–5.52)	60%	9.92, .007
	>0.1 to ≤0.2	656	2.17 (1.94–2.36)	4%		963	4.26 (3.89–4.72)	57%	
	≤0.1	728	2.05 (1.85–2.25)	6%		974	4.02 (3.61–4.33)	55%	
PFS									
Polyclonal IgG levels (for non-IgG myeloma)	>4 to <6	287	1.42 (1.28–1.57)	2%	0.02, .99	367	1.75 (1.55–1.91)	29%	2.91, .23
	≥3 to ≤4	243	1.24 (1.09–1.52)	3%		287	1.80 (1.63–2.07)	31%	
	<3	246	1.35 (1.20–1.54)	1%		389	1.65 (1.42–1.86)	27%	
Polyclonal IgA levels (for non-IgA myeloma)	>0.3 to <0.8	509	1.56 (1.38–1.73)	3%	5.47, .07	832	1.85 (1.69–2.00)	31%	1.24, .54
	≥0.2 to ≤0.3	625	1.52 (1.38–1.65)	2%		402	1.82 (1.59–2.01)	28%	
	<0.2	322	1.61 (1.38–1.79)	2%		734	1.81 (1.65–1.95)	29%	
Polyclonal IgM levels (for non-IgM myeloma)	>0.2 to <0.5	688	1.62 (1.50–1.71)	3%	16.29, .0003	918	1.97 (1.78–2.17)	33%	17.09, .0002
	>0.1 to ≤0.2	655	1.51 (1.36–1.65)	2%		963	1.79 (1.64–1.91)	30%	
	≤0.1	725	1.39 (1.25–1.53)	3%		974	1.71 (1.58–1.82)	27%

In these recent trials, median PFS was 2.83 years in the 342 patients with normal polyclonal IgM levels at diagnosis and 1.80 years in the 2855 patients with IgM levels below the NR. Again, when dividing these 2855 patients into tertiles, median PFS in the lowest IgM tertile group was 1.71 years compared to 1.79 years in the middle group and 1.97 years in the tertile with the least severe immunoparesis (*p* = 0.0002; Table 2 and Fig. 3). Examining the depth of immunoparesis by tertiles for polyclonal IgG and IgA levels at diagnosis showed no significant difference for PFS (*p* = 0.23 and 0.54, respectively). The same was found in the old trials, where the greater the depth of IgM immunoparesis, the shorter the median PFS was while depth of IgG or IgA immunoparesis was not associated with a difference in PFS (Table 2).

It is evident in the Kaplan–Meier curves in Fig. 3 that the survival advantages associated with higher levels of IgM only become apparent after the first year. Analysis of survival data up to 6 months has shown no difference in early deaths between immunoparesis groups. OS was 92% at 6 months for patients with IgM within the NR and 91% in patients with IgM below NR, log rank *p* = 0.65. PFS was 89% within NR and 87% below NR, *p* = 0.21.

Cox regression models were fitted to assess the independent effects of polyclonal IgM on OS and PFS after adjusting for known prognostic factors (age greater or less than 65 years, Sβ2M, BMPCs, ISS and sex) in the older trials with the addition of genetic risk for recent trials, Table 3. These models confirmed polyclonal IgM as an independent prognostic factor of PFS in the old trials (HR = 0.84 (95% CI = 0.73–0.98), *p* = 0.02). Polyclonal IgM was confirmed to be an independent prognostic factor of OS and PFS in the recent trials HR = 0.64 (95% CI = 0.42–0.97, *p* = 0.04) and 0.62 (95% CI = 0.46–0.83, *p* = 0.002), respectively.

**Table 3:** Cox regression table to predict overall survival (OS) and progression-free survival (PFS) from those enroled in MRC myeloma trials from 1980 to 1997 (old trials) compared to the MRC Myeloma IX trial (2003–2008) and the ongoing NCRI Myeloma XI trial (2010–2016)

Old trials (MIV, MV, MVI and MVIII)					Recent trials (MIX and MXI)				
Factor	Grouping	*N*	HR (95% CI)	*p*	Factor	Grouping	*N*	HR (95% CI)	*p*
OS									
SB2M group	≤4, 4–8, >8	1926	1.32 (1.19–1.46)	<.0001	SB2M group	≤4, 4–8, >8	145	1.04 (1.02, 1.06)	.0002
Age group	<65 yrs, ≥65 yrs		1.25 (1.14–1.38)	<.0001	Age group	<65 yrs, ≥65 yrs		1.83 (1.55, 2.17)	<.0001
% BMPCs	<20, 20–50, >50		1.17 (1.09–1.25)	<.0001	% BMPCs	<20, 20–50, >50		1.10 (0.98, 1.23)	.11
ISS	I, II, III		1.18 (1.05–1.32)	.005	ISS	I, II, III		1.32 (1.15, 1.52)	<.0001
Polyclonal IgM	Continuous		0.89 (0.77–1.03)	.12	Polyclonal IgM	Continuous		0.64 (0.42, 0.97)	.04
Sex	Male, female		0.91 (0.83–1.00)	.06	Sex	Male, female		1.04 (0.88, 1.22)	.68
					Genetic risk	SR, HR		1.65 (1.40, 1.95)	<.0001
PFS									
SB2M group	≤4, 4–8, >8	1921	1.21 (1.09–1.33)	.0002	SB2M group	≤4, 4–8, >8	1415	1.03 (1.01, 1.05)	.0002
Age group	<65 yrs, ≥65 yrs		1.13 (1.03–1.24)	.01	Age group	<65 yrs, ≥65 yrs		1.75 (1.54, 1.99)	< .0001
% BMPCs	<20, 20–50, >50		1.23 (1.15–1.31)	<.0001	% BMPCs	<20, 20–50, >50		1.05 (0.96, 1.28)	.33
ISS	I, II, III		1.19 (1.06–1.32)	.003	ISS	I, II, III		1.16 (1.15, 1.51)	.005
Polyclonal IgM	Continuous		0.84 (0.73–0.98)	.02	Polyclonal IgM	Continuous		0.62 (0.46, 0.83)	.002
Sex	Male, female		0.91 (0.83–0.99)	.04	Sex	Male, female		1.13 (0.99, 1.28)	.07
					Genetic risk	SR, HR		1.39 (1.22, 1.58)	<.0001

Polyclonal IgM suppression was shown to be an independent prognostic factor for both OS and PFS when controlling for SB2m, age, BMPCs, ISS, sex and genetic risk in recent trials (*p* = 0.04 and *p* = 0.002, respectively, Table 3). For each point increase in polyclonal IgM, the hazard of death decreased by an estimated 36% and progression or death by an estimated 38%. Within the old trials, IgM immunoparesis was found to be prognostic of PFS independent of Sβ2M, age, BMPCs, ISS and sex, *p* = 0.02.

## Discussion

This study provides in-depth analysis of immunoparesis in newly diagnosed MM patients enroled into UK clinical trials. To our knowledge, this is the first study of this nature in a large cohort of patients enroled in national clinical trials. Findings exposed a notably high incidence of immunoparesis at diagnosis, where, in 80% of patients, polyclonal IgG and IgA immunoglobulin levels were below the NR, and for 89% of patients, polyclonal IgM levels were below the NR. The degree of immunoparesis was also severe, with median polyclonal values presenting substantially below the lower limit of the NRs. The distribution of polyclonal immunoglobulin levels observed in the present analysis also conveys a simple, yet important, clinical message in determining the diagnosis of MM versus other monoclonal gammopathies. If polyclonal immunoglobulins are above the NR, then the patient almost certainly does not have MM. This is supported by none of the 1302 patients without an IgG M-protein being above the NR for polyclonal IgG, and only 8/3226 (0.2%) of patients exhibiting polyclonal IgM levels above NR.

The impact of intact M-protein type on immunoparesis has yielded conflicting findings in previous studies. One study of 940 patients found no significant differences between classes of M-protein [4], whereas another previous investigation of 1027 patients found uninvolved immunoglobulins were reduced in a higher proportion of IgA compared with IgG M-protein patients [2]. We have found the degree of immunoparesis was greatest in patients with IgD and IgA M-protein heavy chain types and with lambda light chain type, but these differences between M-protein types were not large. Similarly, there was a significant but not close association between M-protein levels and degree of immunoparesis. Importantly, LCO patients had severe immunoparesis, indicating that the mechanism of immunoparesis is not dependent on Fc regions of M-proteins.

In the present study, the presence of immunoparesis alone was associated with a negative effect on patient survival and disease control: for each point increase in IgM immunoglobulin levels decreased the risk of death by up to 36%. This is consistent with a previous study, which found preservation of polyclonal immunoglobulins was associated with improved survival in 1755 patients and longer PFS in a smaller sub-cohort of 500 patients [18]. Although, in a more recent Danish multiple myeloma registry study, immunoparesis was associated with shorter PFS, while the shorter OS was not significant in multivariable analysis [31]. The present investigation confirms and extends these findings to a larger population of patients in the context of national clinical trials.

We compared the impact of immunoparesis on survival outcomes in different eras of therapy through assessment of both historical (2608 patients) and recent UK clinical trials (3218 patients). Despite a greater proportion of patients being aged ≥65 years in the more recent MIX and MXI trials, adding predominantly thalidomide or lenalidomide with dexamethasone, patients lived twice as long as the patients from the old trials. This re-confirms the superior efficacy of current anti-myeloma therapies. Importantly, this study highlights preservation of polyclonal immunoglobulin levels of G, A or M class at diagnosis, as a key prognostic factor for OS and PFS in older trials irrespective of age <65 yrs/≥ 65 yrs and with an even more profound effect in recent trials employing modern therapy in both intensive and non-intensive pathways. From old to recent trials, survival has doubled for all patients but the differences in median overall survival times between patients within or below the NR were more pronounced for the new trials (up to 3 years) compared to the old trials (less than 1 year). For patients with normal versus reduced IgM levels, median OS was longer by 29% in old trials and 51% in new trials. Similarly, PFS was longer by 25% in old and 57% in recent trials. This reveals that improved survival from modern therapies has been greatest in patients without severe immunoparesis and that the mechanism of immunoparesis may be an important new therapeutic target.

Over two-thirds of patients had normal levels of polyclonal free light chains and this was not associated with better survival probably because the normal levels were more often the result of reduced glomerular filtration rather than preservation of secretion; 45% of patients had an eGFR <60 mls/min. In recent trials comparing patients with normal polyclonal levels with patients with levels below the NR, median OS and median PFS were significantly longer for patients with normal IgG, IgA or IgM levels. However, only a small proportion of patients had normal immunoglobulin levels (≤20%) and so it was important to see the effect on survival of depth of suppression of polyclonal immunoglobulin levels below the NR. Depth of suppression of polyclonal IgG or IgA levels below the NR was not associated with worse PFS or OS. In contrast, the degree of suppression of polyclonal IgM levels below the NR was significantly associated with survival. In recent trials, the most severely suppressed tertile of patients OS was 0.9 years (18%) shorter than those in the top tertile and 2.57 years (39%) shorter than OS of those with IgM levels within the NR. Similarly, the degree of suppression of polyclonal IgM levels below the NR was significantly associated with worse PFS. The most severely suppressed tertile of patients PFS was 0.26 years (13%) shorter than those in the top tertile and 1.12 years (40%) shorter than PFS of those with IgM levels within the NR.

IgM antibodies are the first class of antibodies produced following antigen exposure, offering early protection against microbial infection and are derived predominantly from secondary lymphoid tissues, not the bone marrow [32]. Consequently, a reduced IgM level in newly diagnosed MM patients has implications for primary antibody response, vaccination efficacy and risk of infection. However, reduced secretion of polyclonal IgM from plasma cells distant from bone marrow also highlights the question of mechanism of immunosuppression and the hypothesis that it might be associated with aggressive MM and reduced immune surveillance of MM.

Infection reaps the greatest toll in mortality in the early months from diagnosis during active disease and we are currently investigating the complexity of that innate and specific immunosuppression in the TEAMM trial that has compared prophylactic levofloxacin antibiotic prophylaxis with placebo. It is clear from the survival curves in Fig. 3 that IgM immunoparesis had little effect on survival during this period while it had a profound effect on overall survival in both old and modern trials. The effect on PFS, in which infection is unlikely to play a major part, is even more interesting. The level of IgM immunoparesis may reflect disease activity/severity and therefore the depth of IgM suppression may be a proxy marker of inherent malignancy and possible resistance to therapy. The mediating factors between low IgM levels and risk of death/disease progression require further investigation and future studies may seek to identify the underlying mechanisms between immunoparesis and patient outcomes. A recent study found recovery of polyclonal immunoglobulins 1 year after autologous stem cell transplantation predicts progression-free and overall survival [33]. Subsequent studies should consider the relative importance of immunoparesis at diagnosis versus post therapy in relation to long-term outcomes.

## Conclusion

The results from this large data set of newly diagnosed MM patients provide a comprehensive up-to-date characterisation of immunoparesis at diagnosis and demonstrate the strong prognostic significance for both progression-free survival and overall survival in historical trials and even more profoundly in present-day clinical trials featuring modern anti-myeloma therapy. In contrast to polyclonal IgG and IgA, it is not simply the presence of immunoparesis but for polyclonal IgM also its severity that predicts patient survival. Immunoparesis is generally thought to reduce survival by increased infection rates but this does not seem to be the predominant mechanism. There is little impact of immunoparesis in the first 6 months from diagnosis the time at which infection is most common. Further immunoparesis profoundly reduces PFS that in itself is little dependent upon infection rates. We hypothesise that IgM immunoparesis impacts survival through a combination of being associated with more aggressive disease and reduced immune surveillance. This impact is much greater in recent trials revealing that improved survival from modern therapies has been greatest in patients without severe immunoparesis and that the mechanism of immunoparesis may be an important new therapeutic target.

## Electronic supplementary material

Supplementary data
