# Supplementary material for: Characterisation of immunoparesis in newly diagnosed myeloma and its impact on progression-free and overall survival in both old and recent myeloma trials
Source: Leukemia. 2018 Jun 20;32(8):1727–38. doi: 10.1038/s41375-018-0163-4 (PMC6087716; doi:10.1038/s41375-018-0163-4)
Supplement: Supplementary file 1 — Supplementary data [file 41375_2018_163_MOESM1_ESM.docx]

**
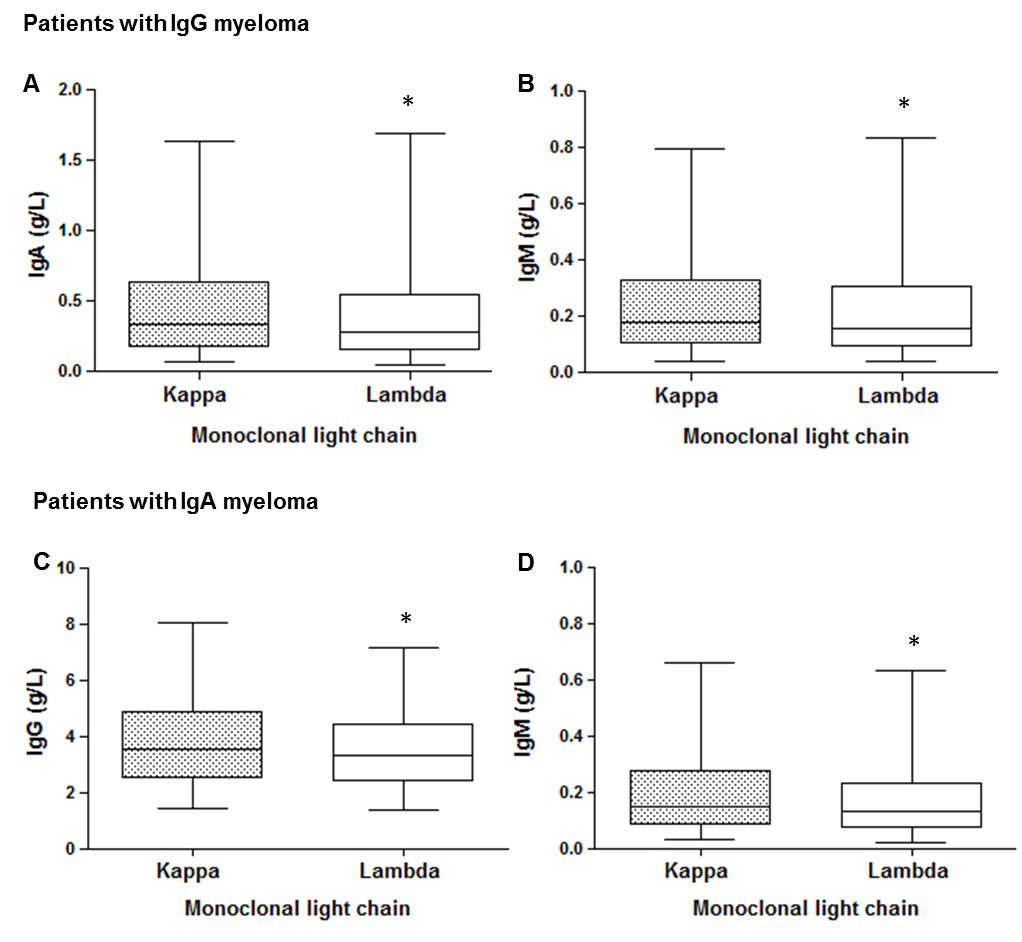
**

**
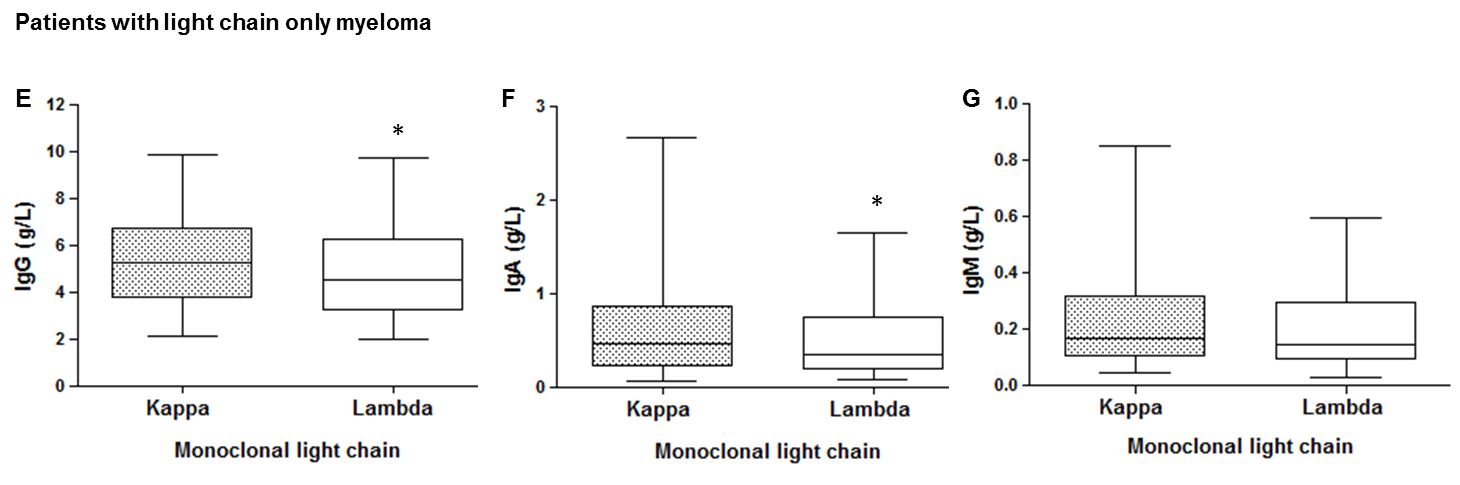
**

**Figure S1**. Comparison of polyclonal immunoglobulins between kappa and lambda light chain patients for the 3 main patient groups: IgG (A and B), IgA (C and D) and light chain only (E, F and G) myeloma. Boxes represent the 25–75^th^ percentile, with the line indicating the median, and whiskers show the 5–95 percentile. Across the groups, lambda patients had significantly lower polyclonal immunoglobulins (with the exception of IgM for light chain only patients); *p* < .05 for all comparisons


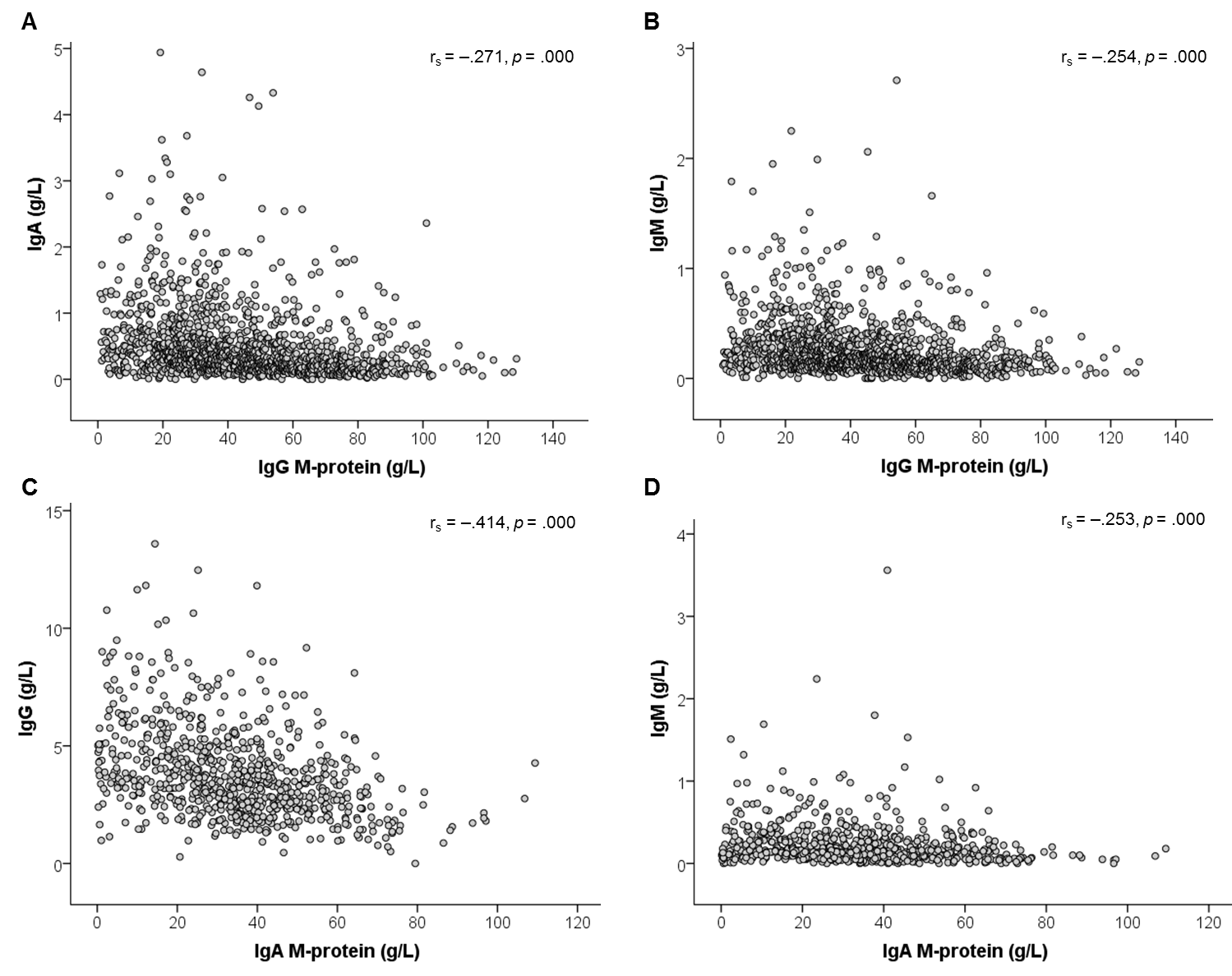


**Figure S2.** Correlations between IgG M-Protein and polyclonal IgA (A) and IgM (B) and between IgA M-Protein and polyclonal IgG (C) and IgM (D).


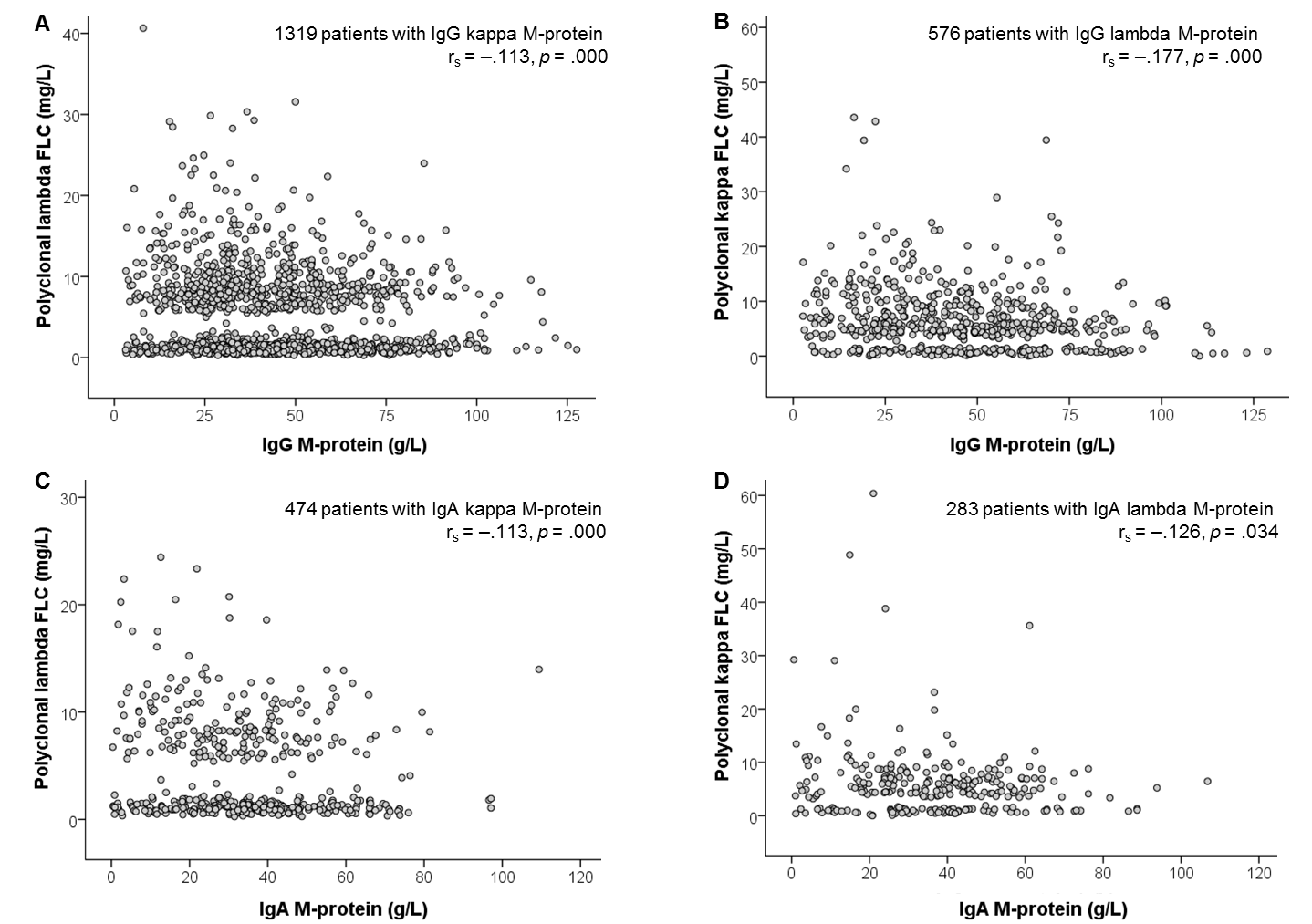


**Figure S3.** Correlations between IgG -protein and polyclonal kappa (A) and lambda (B) serum free light chains (FLC) and IgA M-Protein and polyclonal kappa (C) and lambda (D) serum FLC. Serum FLCs were measured using Freelite.


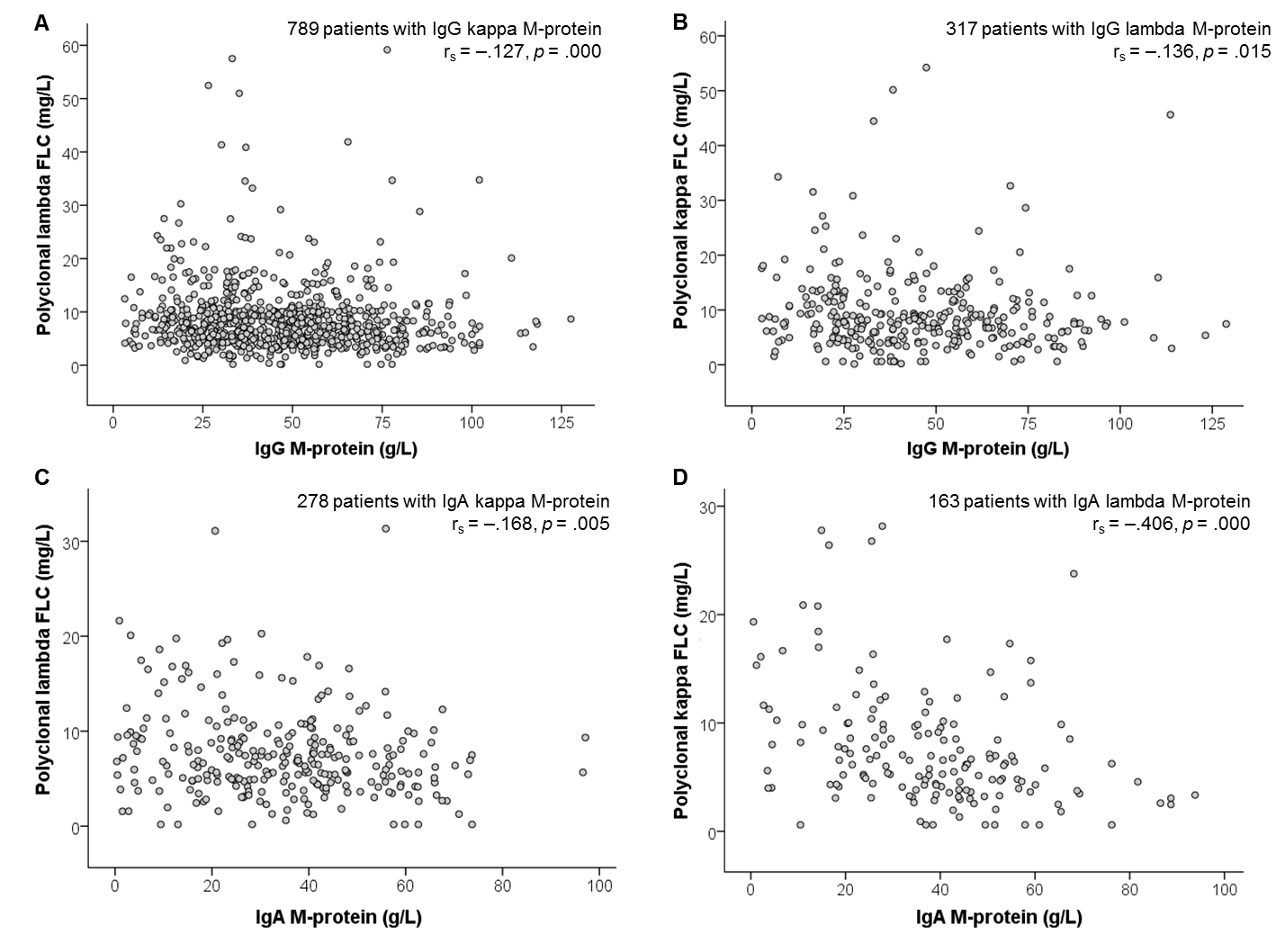


**Figure S4.** Correlations between IgG M-Protein and polyclonal kappa (A) and lambda (B) serum free light chains (FLC) and IgA M-Protein and polyclonal kappa (C) and lambda (D) serum FLC. Serum FLCs were measured using monoclonal anti-free light chain reagents on a Luminex platform

**Supplementary Table 1: Overall survival (OS) and progression free survival (PFS) by M-Protein type**

|  | **Old Trials (MIV, MV, MI and MVIII)** | | | | **Recent Trials (MIX and MXI)** | | | |
| --- | --- | --- | --- | --- | --- | --- | --- | --- |
| **Grouping** | **N** | **Median (95% CI)** | **Censored** | **Logrank x^2^, *p*** | **N** | **Median (95% CI)** | **Censored** | **Logrank x^2^, *p*** |
| **OS** |  |  |  |  |  |  |  |  |
| IgG | 1509 | 2.52 (2.32-2.69) | 6% | 32.42, <.0001 | 1930 | 4.75 (4.41-5.09) | 59% | 7.06, .03 |
| IgA | 718 | 2.33 (2.10-2.56) | 6% |  | 767 | 3.96 (3.62-4.50) | 56% |  |
| Light chain only | 360 | 1.94 (1.56-2.29) | 2% |  | 412 | 4.32 (3.63-5.44) | 57% |  |
| **PFS** |  |  |  |  |  |  |  |  |
| IgG | 1508 | 1.69 (1.62-1.80) | 4% | 24.50, <.0001 | 1930 | 1.89 (1.77-1.98) | 31% | 5.33, .07 |
| IgA | 715 | 1.47 (1.36-1.58) | 3% |  | 767 | 1.71 (1.61-1.89) | 30% |  |
| Light chain only | 359 | 1.46 (1.27-1.59) | 1% |  | 412 | 1.92 (1.65-2.15) | 31% |  |

**Supplementary Table 2:** **Overall survival (OS) and progression free survival (PFS) for patients below the normal range or within the normal range for polyclonal immunoglobulins**

|  |  | **Old Trials (MIV, MV, MI and MVIII)** | | | | **Recent Trials (MIX and MXI)** | | | |
| --- | --- | --- | --- | --- | --- | --- | --- | --- | --- |
|  | **Grouping** | **N** | **Median (95% CI)** | **Censored** | **Logrank x^2^, *p*** | **N** | **Median (95% CI)** | **Censored** | **Logrank x^2^, *p*** |
| **OS** |  |  |  |  |  |  |  |  |  |
| Polyclonal IgG levels | Below normal | 780 | 2.09 (1.86-2.25) | 5% | 7.55, .006 | 1043 | 3.84 (3.44-4.37) | 54% | 15.27, <.0001 |
| (for non-IgG myeloma) | Normal | 370 | 2.48 (2.25-2.74) | 5% |  | 251 | 6.93 (4.89-7.33) | 65% |  |
| Polyclonal IgA levels | Below normal | 1458 | 2.21 (2.06-2.37) | 5% | 22.79, <.0001 | 1968 | 4.42 (4.15-4.82) | 57% | 13.03, .0003 |
| (for non-IgA myeloma) | Normal | 448 | 2.89 (2.62-3.14) | 8% |  | 472 | 6.15 (5.21-7.33) | 65% |  |
| Polyclonal IgM levels | Below normal | 2073 | 2.21 (2.11-2.33) | 6% | 28.60, <.0001 | 2855 | 4.37 (4.14-4.64) | 57% | 16.29, <.0001 |
| (for non-IgM myeloma) | Normal | 534 | 2.85 (2.67-3.21) | 7% |  | 342 | 6.59 (5.29-8.08) | 66% |  |
| Polyclonal kappa free | Below normal |  |  |  |  | 78 | 4.61 (3.07-7.28) | 50% | 0.85, .36 |
| light chain levels  (for lambda patients) | Normal |  |  |  |  | 460 | 4.07 (3.49-4.64) | 48% |  |
| Polyclonal lambda free | Below normal |  |  |  |  | 412 | 5.09 (4.25-6.79) | 58% | 0.33, .56 |
| light chain levels  (for kappa patients) | Normal |  |  |  |  | 752 | 4.75 (4.32-5.63) | 54% |  |
| **PFS** | |  |  |  |  |  |  |  |  |
| Polyclonal IgG levels | Below normal | 776 | 1.36 (1.25-1.46) | 2% | 14.25, .0002 | 1043 | 1.73 (1.62-1.86) | 29% | 21.09, <.0001 |
| (for non-IgG myeloma) | Normal | 370 | 1.67 (1.53-1.86) | 3% |  | 251 | 2.41 (1.96-3.12) | 39% |  |
| Polyclonal IgA levels | Below normal | 1456 | 1.55 (1.45-1.65) | 3% | 22.94, <.0001 | 1968 | 1.83 (1.73-1.93) | 30% | 21.53, <.0001 |
| (for non-IgA myeloma) | Normal | 448 | 1.90 (1.75-2.13) | 6% |  | 472 | 2.49 (2.03-2.79) | 38% |  |
| Polyclonal IgM levels | Below normal | 2068 | 1.51 (1.42-1.58) | 3% | 33.29, <.0001 | 2855 | 1.80 (1.72-1.89) | 30% | 35.22, <.0001 |
| (for non-IgM myeloma) | Normal | 534 | 1.89 (1.77-2.10) | 4% |  | 342 | 2.83 (2.28-3.17) | 42% |  |
| Polyclonal kappa free | Below normal |  |  |  |  | 78 | 1.92 (1.56-2.38) | 19% | 0.004, .95 |
| light chain levels  (for lambda patients) | Normal |  |  |  |  | 460 | 1.78 (1.57-1.95) | 24% |  |
| Polyclonal lambda free | Below normal |  |  |  |  | 412 | 1.97 (1.72-2.20) | 22% | 0.25, .62 |
| light chain levels  (for kappa patients) | Normal |  |  |  |  | 752 | 1.89 (1.68-2.05) | 24% |  |
